# Persistence of Highly Pathogenic Avian Influenza Viruses in Natural Ecosystems

**DOI:** 10.3201/eid1607.090389

**Published:** 2010-07

**Authors:** Camille Lebarbenchon, Chris J. Feare, François Renaud, Frédéric Thomas, Michel Gauthier-Clerc

**Affiliations:** Author affiliations: Centre de Recherche de la Tour du Valat, Arles, France (C. Lebarbenchon, M. Gauthier-Clerc);; Institut de Recherche pour le Développement, Montpellier, France (C. Lebarbenchon, F. Renaud, F. Thomas);; WildWings Bird Management, Haslemere, United Kingdom (C.J. Feare);; Université de Montréal, Montréal, Québec, Canada (F. Thomas)

**Keywords:** Influenza A (H5N1), wild birds, highly pathogenic avian influenza viruses, viruses, influenza, poultry, free-grazing ducks, live bird markets, environment, perspective

## Abstract

Understanding of ecologic factors favoring emergence and maintenance of highly pathogenic avian influenza (HPAI) viruses is limited. Although low pathogenic avian influenza viruses persist and evolve in wild populations, HPAI viruses evolve in domestic birds and cause economically serious epizootics that only occasionally infect wild populations. We propose that evolutionary ecology considerations can explain this apparent paradox. Host structure and transmission possibilities differ considerably between wild and domestic birds and are likely to be major determinants of virulence. Because viral fitness is highly dependent on host survival and dispersal in nature, virulent forms are unlikely to persist in wild populations if they kill hosts quickly or affect predation risk or migratory performance. Interhost transmission in water has evolved in low pathogenic influenza viruses in wild waterfowl populations. However, oropharyngeal shedding and transmission by aerosols appear more efficient for HPAI viruses among domestic birds.

## Abstract 2

Differences in virus evolution may explain virulence heterogeneity.

Wild birds, especially waterbirds of the orders Anseriformes (ducks, geese, and swans) and Charadriiformes (gulls, terns, and waders), are natural hosts for influenza A (avian influenza) viruses. Avian influenza viruses are classified on the basis of genetic, antigenic, and structural characteristics of hemagglutinin and neuraminidase proteins. These proteins are involved in binding of virus to host cells and release of new virions from these cells, respectively. Sixteen hemagglutinins (H1–H16) and 9 neuraminidases (N1–N9) have been described. For avian influenza viruses of subtypes H5 and H7, there are 2 types of virulence: low pathogenic avian influenza (LPAI) virus generally produces benign intestinal tract or respiratory infections; highly pathogenic avian influenza (HPAI) virus generally produces multiorgan systemic infections.

LPAI viruses naturally infect wild waterbirds according to host species, age, immune status, feeding behavior, premigration aggregation, and aquatic survival of the virus. Long-term studies in Europe and North America also identified seasonal variation in prevalences of infection of LPAI virus and circulating subtypes. HPAI viruses primarily infect poultry in which viruses of subtypes H5 and H7, presumably from wild birds or contact with their derivatives, sporadically switch to highly virulent strains.

At the end of the 19th century, a disease that caused high mortality rates and spread rapidly was described in domestic birds in Italy. This fowl plague spread through Europe in the early 20th century, most likely through trading of domestic birds. In 1955, the pathogen responsible for the disease was classified as an influenza A virus, and its relationship to human influenza viruses was recognized. Domestic birds have been affected by recurrent outbreaks of HPAI viruses, generally limited to localized geographic areas but responsible for high mortality rates and substantial economic losses. In contrast, wild birds have rarely been involved in HPAI virus infections. Before 1996, only 1 HPAI virus outbreak was documented in the wild, resulting in the death of ≈1,300 common terns (*Sterna hirundo*) in South Africa (*1*). Since then, emergence and spread of the HPAI virus lineage from Asia (H5N1), first discovered in domestic geese in southern People’s Republic of China in 1996, has been responsible for the death of thousands of wild birds, occasionally through mass mortality events (e.g., Lake Quinghai, People’s Republic of China, in May–June 2005). Extensive surveillance of apparently healthy wild populations has rarely detected HPAI virus (H5N1), even in areas where the virus is endemic in domestic birds (*2*). In addition, some reports of asymptomatic infection by HPAI virus (H5N1) in apparently healthy free-living wild birds lack important substantiating information and such cases of infection have yet to be convincingly demonstrated (*3*).

Although recent studies have focused on environmental factors that contributed to the persistence and spread of HPAI virus (H5N1) in southeastern Asia, Europe, and Africa (*4**–**6*), general knowledge concerning mechanisms of emergence and persistence of HPAI viruses is limited. We propose that because the ecologic landscape in which avian influenza viruses evolve differs markedly between natural (i.e., wild birds) and artificial (e.g., intensive poultry farming, free-grazing ducks, and live bird markets) conditions, selective pressures differ. These phenomena are likely to explain virulence heterogeneity among avian influenza viruses and why HPAI viruses do not naturally emerge or persist in natural ecosystems.

## Natural Selection

The avian influenza virus genome is composed of 8 segments of negative single-stranded RNA coding for 11 proteins. Replication by these viruses is termed low fidelity because RNA mutations, due to imprecision in the replication processes, lead to a wide diversity of genetic variations in progeny. Genetic reassortment between segments of different virus subtypes during co-infection of a host cell further contributes to progeny diversity, providing a basis for rapid evolution and emergence of new avian influenza viruses in the wild (*7*). The switch from an LPAI virus to an HPAI virus phenotype is achieved mainly by introduction of multiple basic amino acid residues into the hemagglutinin cleavage site. This introduction generally occurs in poultry, as has been demonstrated experimentally (*8**,**9*).

Transmission–virulence trade-off models proposing that high rates of pathogen transmission indirectly select for higher levels of virulence have long dominated scientific thinking. Along with recent criticisms of these simplistic models (*10*), we consider that differences in host conditions and environments are also major determinants of virulence evolution. In many host-pathogen interactions, evolution toward an optimal virulence can occur, more or less rapidly, after successful introduction into a new host species (*11*). Virulence evolution involving the occasional switch from an LPAI virus to an HPAI virus, after introduction into domestic birds (*12*), does not solely result from host species switch but is probably driven by a larger set of ecologic parameters encountered in artificial ecosystems. These ecosystems include poultry farming (especially when intensive), free-grazing duck production, and live bird markets. Thus, it is likely that LPAI viruses and HPAI viruses are adapted respectively to natural and artificial ecosystems in which they face different ecologic constraints such as host population structure, density and genetic diversity, and optimal opportunities for virus transmission (Figure).

**Figure:**
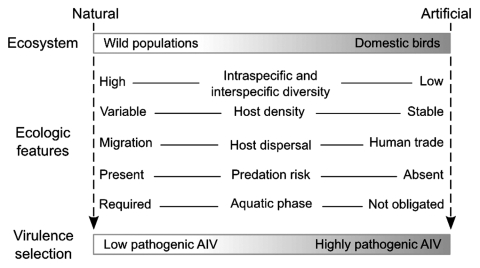
Comparison of natural versus artificial ecosystems showing different ecologic constraints for evolution of avian influenza virus (AIV).

Under the conditions in which domestic birds are maintained, the range of host species available for infection, compared with natural ecosystems, is considerably reduced and limited mainly to galliform birds and waterfowl, often in monospecific flocks. Poultry host density is often considerably higher than the virus would encounter in the wild, and in intensive systems the high density is maintained throughout the life of the flock. Age structure is generally more uniform and environmental conditions are frequently kept more equable and constant. In addition, wider opportunities for viral transmission exist in the form of multiple physical transport mechanisms for living poultry and their products. The latter mechanisms include feces, feathers, and meat, and physical transport can include cages, packaging, farm workers and their clothes, and vehicles used on farms and over long distances. During these physical modes of transport, the ability of the virus to survive in the various environments encountered is likely to be subject to selection, but at this stage pathogenicity per se to potential hosts will assume no major role, unless this also affects environmental survival. Under these circumstances, selective pressures differ greatly from those encountered by the virus in their natural, primarily aquatic, ecosystems. Avian influenza virus strains that have evolved to survive under these domestic conditions are highly likely to be maladapted to natural ecosystems and hosts. In particular, HPAI viruses, which often induce high and rapid lethality in their hosts, require high and sustained host contact rates that are rare under natural conditions, being restricted to extreme weather conditions or to particular stages of the life cycle.

HPAI viruses in wild birds appear to retain their high virulence, leading to infections being discovered almost entirely in sick or dead birds (*13*). After HPAI virus outbreaks in wild birds, LPAI viruses with genetic affinities to HPAI virus lineages have not been reported. Although there have been examples of mutual host–pathogen co-evolution when new highly virulent viruses enter new wild hosts, e.g., amelioration of myxomatosis virulence in wild rabbit (*Oryctolagus cuniculus*) populations (*14*), there is no indication that co-evolution occurs when HPAI viruses gain access to wild bird populations.

## Host Density and Diversity

The influence of host population structure in selection for virulence is often critical (*15*). Levels of virulence are, in part, determined by and proportional to the frequency with which interhost transmission opportunities occur (*10*). Low virulence can be selected for when host–host contacts are infrequent, and high virulence can be selected when the host contact rate is high (*16*). In wild bird populations, contact rates between individuals differ markedly between seasons (e.g., reproductive period, molt, migration, wintering), species (e.g., colonial birds), and age. High densities of birds are reached by some species in molting and wintering areas. However, these seasonal aggregations do not lead to selection and emergence of HPAI viruses. Compared with conditions in the wild, host densities in farming conditions are not only high (even extreme under intensive poultry production), but consistently so, which may be a major determinant for selection of high virulence.

In the wild, in addition to variable host contact and thus transmission rates, avian influenza viruses encounter high host-species diversity, as in multispecies waterfowl aggregations. In this context, generalist pathogens are probably favored because they can infect a large spectrum of host species, thereby maximizing replication and dispersal opportunities (*17*). LPAI viruses have been isolated from >105 bird species in 26 families (*13*), suggesting that they are able to infect a large diversity of host species. Under farming conditions, the low (sometimes null) host species diversity is likely to select for species-specific or ecosystem-specific pathogens.

Recently, some studies investigated species-related variation in susceptibility (from ducks to passerine birds) and clinical signs generated by infection with HPAI virus (H5N1). Among the bird species artificially challenged with virus, some laboratory-maintained mallards (*Anas platyrhynchos*) did not show clinical or pathologic signs related to HPAI virus (H5N1) infections (*18**,**19*). Variations related to virus excretion have been reported between studies, but 1 study reported that the domestic mallard can excrete HPAI virus (H5N1) for long periods (<17 days depending on the virus strain) (*20*). Such a long duration contrasts with LPAI virus excretion recently reported in wild mallard populations, in which virus was shed for <8 days with a mean minimum duration of only 3 days (*21*). The domestic mallard could act as an efficient host reservoir in domestic birds and favor viral transmission during an extended period, without clinical evidence of infection. Such evolution of host specificity is believed to have contributed to the spread and endemicity of HPAI viruses (H5N1) in Asia through domestic ducks (*20**,**22**,**23*).

Similarly, LPAI viruses must evolve in the face of intraspecific variability between individuals of a host population. In the wild, immune response varies among birds of the same species, depending on their genetic backgrounds, age, breeding, molting, migration, health status, and exposure to previous infections with pathogens and other parasites (*24*). LPAI viruses are adapted to this diversity in wild birds. Conversely, under many modern farming conditions, potential hosts have low genetic diversity and highly structured age distribution and are regularly protected from some pathogens through vaccination and antimicrobial drugs. Such artificial conditions are likely to select for specialist pathogens. Knowledge on the effects of intraspecific diversity on the evolution of avian influenza viruses is limited to extrapolations from experimental studies. However, these experimental conditions do not reflect intraspecies diversity encountered in the wild and avoid detection of potential individual variation in response to avian influenza virus infection, according to host life history traits. Development of experimental and theoretical studies focusing on the effects of host density and diversity at the community and species levels and in different environments (ranging from totally enclosed to open air) should provide useful information regarding evolution of virulence of avian influenza viruses in natural and artificial ecosystems.

## Should I Kill My Host?

In many host–pathogen associations, the pathogenesis related to infection leads to an overall weakening of infected individuals. Although LPAI virus infections in wild birds are generally considered to be benign, in poultry they can cause mild disease, depression, and problems with egg production (*25*). However, physiologic and behavioral effects in wild birds may have been overlooked. van Gils et al. (*26*) reported impaired foraging and migration efficiencies in infected Bewick swans (*Cygnus columbianus bewickii*), suggesting that host behavior might be affected by LPAI viruses in subtle ways not previously envisaged. Latorre-Margalef et al. (*21*) also reported that body mass was lower in infected wild mallards than in uninfected wild mallards and that the amount of virus shed by infected juveniles was negatively correlated with body mass. These recently discovered effects of LPAI virus infection, although mild, could nevertheless have implications for host fitness. For example, delayed migration and impaired foraging of Bewick swans likely retarded their arrival on their arctic breeding grounds, reducing the chances of these birds reproducing successfully in the year of infection. The implications of such mild symptoms for transmission and evolution of avian influenza viruses remain to be determined.

Given that predators sometimes prey preferentially on sick animals, differential susceptibility between several host species (or individuals) can lead to greater predation on the more susceptible ones (*27*). Adverse physiologic or behavioral effects of infection might decrease host antipredator performance, favor predation on infected birds, and thus decrease transmission of avian influenza viruses among target hosts of the pathogen (i.e., immunologically unexposed waterbirds). Conversely, host antipredator performance reduction can favor infection of predators, and host fatality can lead to infection in scavengers. Such virus transmission between prey and predators and scavengers has been shown for HPAI virus (H5N1), with reported deaths in crows, birds of prey, and mammals (e.g., Felidae and Mustelidae). However, transmission of avian influenza viruses has not been sustained within predator and scavenger populations, indicating that these host infections represent a dead-end for virus transmission. Viruses inducing strong physiologic effects, even if they do not directly lead to death, are therefore unlikely to be selected in wild waterbird populations if they affect host antipredator performance. Studies focusing on differential fitness and predation rates on avian influenza virus–infected wild bird species, considering the effects of the infection itself, but also differences in behavior induced by infection, would help to clarify these aspects. In terms of fitness, experiments on artificially infected captive waterfowl, examining egg mass, clutch size, incubation behavior, hatching success, chick mass, and chick survival could also be illuminating.

Pathogen distributions and abundances in the environment can alter the decisions hosts make with regard to whether to stay and resist or to disperse from 1 place to another with lower pathogen risk (*28*). Pathogen transmission and dispersal are intricately linked, and it is widely acknowledged that pathogens may benefit from investing in dispersal strategies. Dispersal of avian influenza viruses is poorly documented in natural conditions. For HPAI viruses, current knowledge of the potential for virus dispersal through long-distance migration is mainly limited to extrapolations from experiments on captive-reared ducks performed under laboratory conditions (*19*). These birds are not subjected to sustained high-energy expenditure and unlikely to experience immunosuppression, for which there is increasing evidence in birds undertaking migration (*29*). Although infected birds might be able to disperse virus over short distances, e.g., during periods of cold weather (*4*), experiments in which birds are subjected to physiologic stresses associated with migration are needed to determine their capacity to spread virus over long distances. Experimental studies with captive wild waterbirds could test responses to infection during exposure to realistic physiologic or nutritional stresses that replicate long-distance migration or winter food shortage. In addition to monitoring the extent of virus shedding, effects on physical activity, response to stimuli, or time spent feeding should be investigated. Indirect estimations of virus dispersal derived from knowledge of bird migrations could also provide complementary information related to the spread of avian influenza viruses.

## Life Outside the Host

Transmission of LPAI viruses among wild waterbirds is considered to be mainly by the fecal–oral route, with virus particles excreted from infected birds directly from feces into water and contracted by potential hosts by ingestion of virions in water or on food therein (*30*). Although no evidence has been provided, potential fecal–fecal transmission through fecal drinking could also favor infection of the cloaca and the lower part of the digestive system. Recent studies have highlighted that HPAI virus (H5N1) replicates more (and for longer periods) in the host bird trachea than in the digestive tract (*18**,**19*). In addition, severe lung congestion and alveolar and bronchiolar edema, together with virus isolation from tracheal swab specimens, suggested that oropharyngeal excretion occurred in infected wild birds (*31*). Preponderance of oropharyngeal excretion is associated with systemic infections caused by HPAI viruses, in contrast to the propensity for cloacal virus excretion associated with digestive tract infections of LPAI viruses. Thus, oropharyngeal and fecal excretion represent 2 strategies that may be selected according to ecosystem characteristics. Production of viral particles in aerosols is probably the most efficient transmission strategy in confined environments with high densities of birds, high ambient temperature and humidity, and forced air circulation, as under intensive farming conditions (*32*). Selection for systemic infection, accompanied by oropharyngeal excretion and airborne transmission, could potentially be favored under these circumstances (*33*). Experimental studies focusing on the evolution of wild-origin LPAI viruses in domestic birds in confined environments could provide interesting insights regarding selection for oropharyngeal excretion and airborne transmission.

However, after excretion, virus must survive in the environment long enough to be able to contact and infect susceptible hosts. Although persistence of avian influenza viruses in water appears to be the natural mechanism to maintain and transmit influenza viruses in wild bird populations, Brown et al. (*34*) compared 2 strains of HPAI virus (H5N1) with several wild bird–origin LPAI viruses and found that HPAI virus (H5N1) does not persist in water as long as LPAI viruses, at least under experimental conditions. This finding suggests that HPAI viruses could be less adapted than LPAI viruses to spread by the fecal–oral route in water. Inactivation processes of avian influenza viruses in the environment are far from being well understood, but abiotic factors such as salinity, temperature, relative humidity, or ultraviolet radiation are likely to play a key role (*33**,**35*).

## Avian Influenza Virus in a Human-Made World

Technologic and cultural changes in human populations open new ecologic niches for pathogens, which differ from niches available in the wild, and inevitably influence their evolution (*36*). Networks of poultry production are likely to favor persistence of virulent strains, with continuous circulation of avian influenza viruses between connected farms or markets. Such networks probably favor the endemicity of HPAI virus (H5N1) in Southeast Asia (*6*). Multispecies live-animal markets are good examples of how humans have artificially created a dynamic system in which a large variety of avian influenza viruses can be generated and maintained, thus offering enhanced opportunities for genetic reassortments (*37*). Connectivity in modern human populations through transportation has increased during the past century, especially during the past few decades, in volume and, with regard to virus spread, in speed and geographic extent. The past 2 decades have seen a huge increase in poultry production and associated national and international trade in Southeast Asia. After adapting to intensive farming processes, avian influenza viruses can be spread intercontinentally among domestic bird populations by human activities. This finding appears to be the most likely scenario for spread of HPAI virus (H5N1) from Asia to Europe and Africa, in which the poultry trade (legal, unregulated, and illegal) seems to have been the predominant mechanism (*38*).

Thus, human activities are likely to artificially shape evolutionary ecology of avian influenza viruses and select for traits (e.g., virulence, oropharyngeal excretion, host specialization) that confer optimal viral fitness under the artificial conditions of poultry production, distribution, and processing. Evolution of this host–pathogen system created by humans might represent one of the main threats to human health. Because of an increasing number of studies focused on genetic characteristics of avian influenza viruses, we are aware of the mechanistic basis of high pathogenesis. However, our effort to predict and control emergence of these viruses through this complex host–pathogen system must consider host ecology and ecosystem characteristics (natural or linked with human activities) in which these viruses evolve.

If application of evolutionary theory to medical sciences enables a predictive framework for long-term host–pathogen interactions, it also provides interesting possibilities for design of medical and public health protection measures (*39*). Integration of ecologic and evolutionary theory in epidemiology and human diseases has shown increased interest (*40*). These theories could provide useful information for long-term disease management. However, such approaches and their possible applications for avian influenza viruses are lacking.

Do we have to favor developments of poorly diversified farming conditions with high densities of genetically impoverished birds? What are the long-term effects of mass vaccination? Can we avoid virus exchanges between wild and domestic birds? Answering such key questions first requires sound understanding of natural mechanisms of virulence selection and, from that knowledge, taking account of ecologic features that may select for HPAI viruses in artificial ecosystems.
